# Trends in the Frequency of HLA DR-DQ Haplotypes Among Children and Adolescents with Type 1 Diabetes Mellitus in the Southeast Region of Turkey

**DOI:** 10.4274/Jcrpe.768

**Published:** 2012-12-19

**Authors:** Mehmet Keskin, Ayşe Aygün, Sacide Pehlivan, Özlem Keskin, Yılmaz Kor, Ayşe Balat, Yavuz Coşkun

**Affiliations:** 1 Gaziantep University Faculty of Medicine, Department of Pediatric Endocrinology and Metabolisms, Gaziantep, Turkey; 2 Gaziantep University Faculty of Medicine, Department of Pediatrics, Gaziantep, Turkey; 3 Gaziantep University Faculty of Medicine, Department of Medical Biology, Gaziantep, Turkey

**Keywords:** type 1 diabetes mellitus, HLA DR-DQ haplotype, allele, children, Autoimmunity

## Abstract

**Objective:** The aim of this study was to determine the frequency of HLA DR-DQ haplotypes in children with type 1 diabetes mellitus (T1DM) in the Southeast Region of Turkey.

**Methods:** Eighty children and adolescents with T1DM and eighty control subjects participated in the study. HLA-DR, DQ was typed using polymerase chain reaction and sequence-specific priming technique.

**Results:** HLA DRB1*03 allele was significantly more common in patients than in control subjects. HLA DRB1*11, HLA DRB1*13 and HLA DRB1*14 allele frequencies were significantly lower in patients than in controls. DQB1*02 allele was more common in patients, whereas DQB1*03 allele was more frequent in control subjects. HLA DRB1*03-DQB1*02 haplotype was more frequently observed among patients.

**Conclusion:** These results confirm the similar potential trends in the frequency distribution of HLA susceptibility genes with T1DM previously observed in Turkey and in other Caucasian populations.

**Conflict of interest:**None declared.

## INTRODUCTION

Type 1 diabetes mellitus (T1DM) is a chronic autoimmune disease caused by deficiency in insulin secretion. The incidence of T1DM rises faster than expected (1). Etiology of T1DM is complex, and both genetic and environmental factors play important roles. T1DM is an autoimmune disorder and the risk for the disease is increased by specific HLA DR/DQ alleles [e.g., DRB1*03-DQB1*0201 (DR3) or DRB1*04-DQB1*0302 (DR4)] (2,3,4,5). Trends in the frequency of HLA DR-DQ haplotypes showed different associations in distinct ethnic groups (6,7,8,9,10,11,12,13,14,15) and also in the same region (11). With these variations in mind, T1DM patients and healthy controls from the population of the Southeast Region of Turkey were investigated for HLA DR-DQ haplotypes. 

## METHODS

Eighty unrelated children and adolescents with T1DM (41 boys and 39 girls, mean age 10.8±3.4 years) attending the Pediatric Endocrinology and Metabolism Unit of Gaziantep University Faculty of Medicine and eighty healthy children and adolescents (48 boys and 32 girls, mean age 10.5±2.2 years) participated in the study. Gaziantep is a city with a population of 1 390 000 in the Southeast Region of Turkey and our Unit serves as a reference center for the region. Patients with other forms of diabetes, such as T2DM, maturity-onset diabetes of the young, thiamine-responsive megaloblastic anemia, were not included in the study. All patients included in the study were receiving insulin for controlling their hyperglycemia. The study received approval from the ethics committee of the hospital. After the patients and control subjects signed an informed consent form, blood samples were collected. Total genomic DNA was extracted from ethylenediaminetetraacetic acid-anticoagulated venous blood by a modified ‘salting out’ technique, precipitated with ethanol, and resuspended in sterile distilled water to a final concentration of 0.1-1.0 μg/ μL before use. HLA-DR, DQ was typed using polymerase chain reaction and sequence-specific priming (SSP) technique using the SSP2L HLA class II genotyping kit. 

## STATISTICAL ANALYSIS

Analysis was performed using SPSS version 11 software for Windows. Data are presented as means ± SD (range). Differences in frequencies for categorical variables were assessed by the chi-square test. We compared the groups using the independent-samples t-test. A p-value of less than 0.05 was considered statistically significant in all data analyses. 

## RESULTS

Male/female ratio was 41/39 in the patient group. The mean age of the group was 10.8±3.4 years. The mean age at disease onset was 8.0±3.5 years. At disease onset, the mean HbA1c level of the patients was 11.07±2.7 % and their mean C-peptide level was <0.5 ng/mL. Male/female ratio was 48/32 in the control group. The mean age was 10.5±2.2 years. There were no statistically significant differences in age and sex distribution between the diabetic and control groups.

HLA DRB1*03 allele frequency was significantly higher in patients than in control subjects, while the frequencies of HLA DRB1*11, HLA DRB1*13 and HLA DRB1*14 alleles were significantly lower in patients than in controls (Table 1). DQB1*02 allele was significantly more common in patients, whereas DQB1*03 allele was more frequent in controls (Table 2). The frequency of HLA DRB1*03-DQB1*02 haplotype was significantly higher among patients than controls (Table 3). 

## DISCUSSION

The incidence of T1DM is rapidly increasing in specific regions and shows a trend toward earlier age of onset and also it is highly variable among different ethnic groups (6,7,8,9,10,11,12,13,14,15). It is predicted that the overall incidence of T1DM is about 40% higher in recent years as compared to its incidence in 1997 (16).

The genetic background of T1DM is polygenic with the major disease locus located in the HLA region. Mapping studies in T1DM have identified T1DM interval at chromosome 6p21 as the first major susceptibility marker, which also contains the MHC class II region (2,3,4,5). However, the extensive polymorphism and the functional relevance of MHC genes to an immune response have resulted in many population-based association studies. HLA-DR3 and -DR4 were shown to be strongly associated with the disease in Caucasoid populations (5,6,7). HLA-RB1 and DQB1 contribute to the genetic susceptibility of T1DM, and they are involved in the induction of the autoimmune destruction of pancreatic beta-cells precipitating the disease. Many studies have shown associations not only for DRB1 and DQB1, but also for DQA1 and DPB1 alleles with T1DM. The highest risk for developing the disease has been associated with heterozygous DR3/DR4 phenotype, particularly in combination with DQA1*0301-DQB1*0302 alleles (2,3,4,5). Many different negative associations with T1DM were also reported, DRB1*1501 DQA1*0102 DQB1*0602 being the strongest (17). The major genetic determinants of T1DM are alleles at the HLA-DRB1 and DQB1 loci, with both susceptible and protective DR-DQ haplotypes present in all human populations. The HLA-DR and -DQ genes are well established as being associated with increased risk for T1DM. The lowest incidence of childhood T1DM in Europe has been reported from the Republic of Macedonia (15). Similar disease associations were found in other Caucasian populations. HLA-(DR3)-DQA1*05-DQB1*02 was the most common disease-associated haplotype, but several DRB1*04-DQB1*0302 haplotypes were also found to be increasing among T1DM patients. T1DM incidence rates are extremely low in Asian populations (18). The prevalence of islet-specific autoantibodies is reported to be low compared with Caucasians. In Japanese patients with "classic" T1DM, DRB1*0405-DQB1*0401 and DRB1*0901-DQB1*0303 are major susceptible HLA-DR-DQ haplotypes, whereas DRB1*1502-DQB1*0601 and DRB1*1501-DQB1*0602 are protective (8). DRB1*0405-DQB1*0401, DRB1*0802-DQB1*0302, DRB1*0901-DQB1*0303 and DRB1*1302-DQB1*0604 haplotypes were found to play a significant role in the etiology of the disease. DRB1*1501-DQB1*0602 haplotype was identified as a protective. Caucasians and Asians have shown differences in the frequency of the haplotype. The incidence of DR13 in an elderly Japanese population with T1DM was significantly higher (8). Sardinia is the area with a very high incidence rate for T1DM (19). DRB1*405-DQA1*0301-DQB1*0302 haplotype confers strong susceptibility to T1DM in Sardinia people, and also in Asian people such as the Chinese and Koreans (18,19). It is certain that DR-DQ linkage disequilibrium is an important factor explaining the difference in T1DM incidence in different countries (20). DRB1*0301-DQB1*0201 and DRB1*1307-DQB1*0302 haplotypes in the Arabs with T1DM in Lebanon and DRB1*0401-DQB1*0302 haplotypes in the Arabs with T1DM in Bahrain were higher (9). DRB1*0401-DQB1*0302 haplotypes were significantly greater in patients with T1DM. T1DM-predisposing haplotype DRB1*0301-DQB1*0201 is similar to that in our study and Caucasians. In a study covering the years 2002-2004, DRB1*04-DQB1*03 haplotype was found to be more frequent in Colorado youth with T1DM (11). HLA DRB1*0301-DQB1*0201 haplotype was high in the North African immigrants and French natives (13). In a study of the Israeli population with T1DM, DRB1*03-DQB1*02/DRB1*04-DQB1*03 genotype was found to be more frequent (14).

Genes for T1DM may provide both susceptibility to and protection from the disease.

DRB1*03-DQB1* 02 and/or DRB1*04-DQB1*03 haplotypes were associated with increased susceptibility to T1DM in Caucasians, while DRB1*15-DQB1*06 haplotype was shown to play a prophylactic role (17). In this study, HLADRB1*03-DQB1*02 haplotype was mostly found in T1DM similar to Caucasians with T1DM. Saruhan-Direskeneli et al (12) had investigated the relationship between T1DM and HLA class 2 in Turkey previously. The results of this study were similar to those of our study - DRB1*03-DQB1*02 haplotype was found frequently in patients with T1DM. However, the authors reported that haplotypes including DRB1*04 allele also increased the incidence of T1DM, a finding which we did not observe. The most common combinations found were of DRB1*04O2-DQB1*0302 haplotype. In this study, T1DM-protective haplotypes were identified: DRB1*1401-DQB1*0503, DRB1*1303-DQB1*0301, DRB1*1502-DQB1*0601, DRB1*1301-DQB1*0603, DRB1*1101-DQB1*0301, DRB1*1301-DQB1*0603, DRB1*0701-DQB1*02. In addition, combinations of DQA1 alleles were also specified. There were no protective haplotypes identified as significant in our study.

In our study, HLA DRB1* allele distribution of HLA DRB1*03 patients was shown in 50 patients (62.5%) and it was statistically significant. HLA-DRB1*04 allele was observed in 20 patients (25%). These results are in compliance with published data.

DRB1*08 allele was found in patients with T2DM (10). It is encountered less frequently in T1DM. Only 3 children (3.8%) with DRB1*08 allele were found in this study and they were from the control group.

In conclusion, we found that HLA DRB1*03-DQB1*02 haplotype was more common among our T1DM patients. These results are in agreement with the frequency distribution of HLA susceptibility genes in T1DM patients previously observed in Turkey and other Caucasian populations.

## Figures and Tables

**Table 1:**
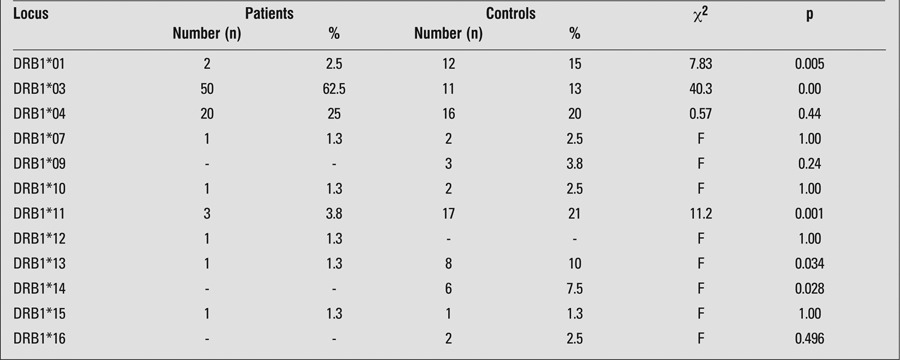
Distribution of HLA-DRB1* alleles

**Table 2:**
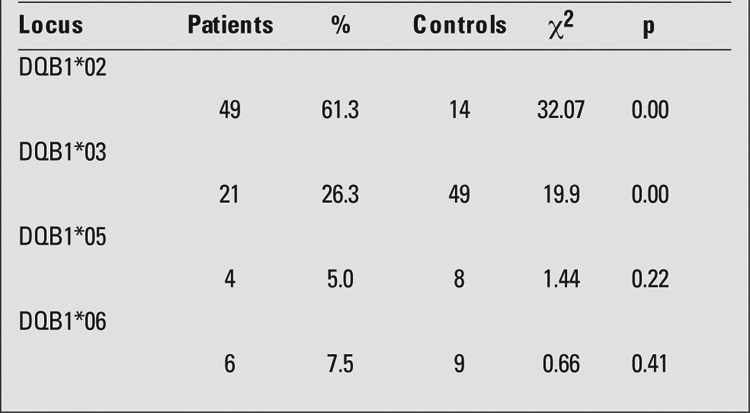
Distribution of HLA-DQB1* alleles

**Table 3:**
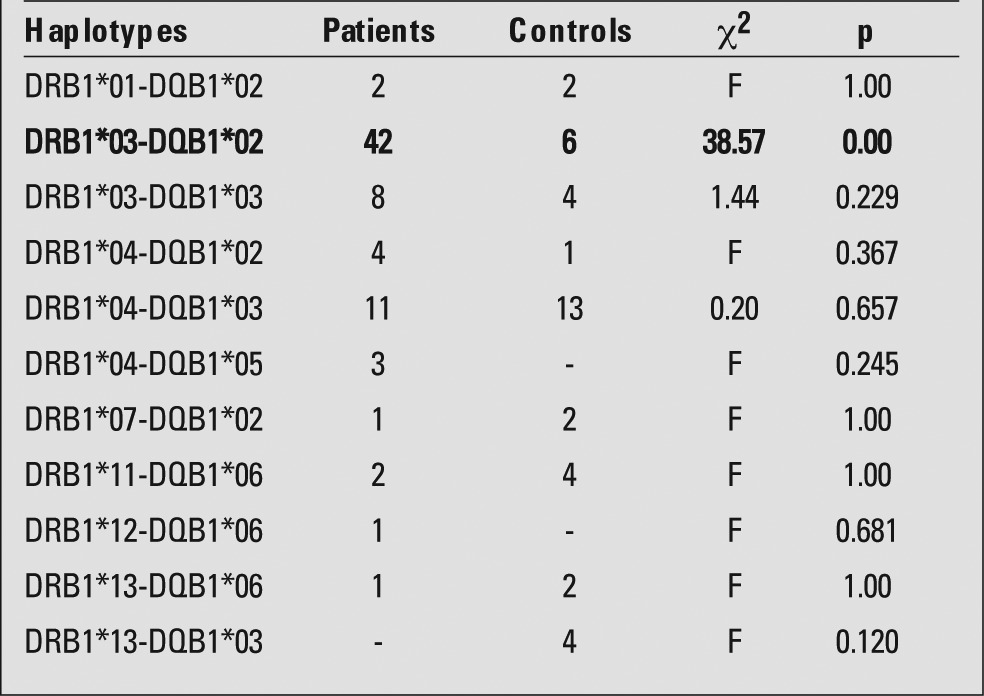
Distribution of HLA DR/DQ haplotypes
